# Therapeutic role of exosomes and conditioned medium in keloid and hypertrophic scar and possible mechanisms

**DOI:** 10.3389/fphys.2023.1247734

**Published:** 2023-09-12

**Authors:** Yixiu Zhong, Youfan Zhang, Aijiao Yu, Zhiwen Zhang, Zhenjun Deng, Kaifen Xiong, Qi Wang, Jianglin Zhang

**Affiliations:** ^1^ Department of Dermatology, Shenzhen People’s Hospital (The Second Clinical Medical College, Jinan University, The First Affiliated Hospital, Southern University of Science and Technology), Shenzhen, Guangdong, China; ^2^ Department of Dermatology, Nanfang Hospital, Southern Medical University, Guangzhou, China; ^3^ Department of Dermatology, Dermatology Hospital, Southern Medical University, Guangzhou, China; ^4^ Department of Dermatology and Venereology, Nanfang Hospital, Southern Medical University, Guangzhou, China; ^5^ Department of Dermatology, Xiangya Hospital, Central South University, Changsha, Hunan, China

**Keywords:** exosomes, conditioned medium, mesenchymal stem cell, keloid, hypertrophic scar

## Abstract

Exosomes, ranging from 40 to 160 nm in diameter, are extracellular lipid bilayer microvesicles that regulate the body’s physiological and pathological processes and are secreted by cells that contain proteins, nucleic acids, amino acids and other metabolites. Previous studies suggested that mesenchymal stem cell (MSC)-derived exosomes could either suppress or support keloid and hypertrophic scar progression. Although previous research has identified the potential value of MSC-exosomes in keloid and hypertrophic scar, a comprehensive analysis of different sources of MSC-exosome in keloid and hypertrophic scar is still lacking. This review mainly discusses different insights regarding the roles of MSC-exosomes in keloid and hypertrophic scar treatment and summarizes possible underlying mechanisms.

## 1 Introduction

### 1.1 Mesenchymal stem cells (MSCs) and conditioned medium

#### 1.1.1 Mesenchymal stem cells (MSCs)

MSCs are the most widely studied stem cells, featuring self-renewal and multilineage differentiation capacity. MSCs are generally classified according to their source: bone marrow, trabecular bone, adipose tissue, synovium, skeletal muscle, lung, deciduous teeth, and human umbilical cord (Baksh et al., 2004). Different sources of MSCs possess similar morphological and biological features. MSCs have displayed promising potential in immune modulation, higher proliferation, tissue regeneration and angiogenesis (Hoang et al., 2022). It was suggested that human umbilical cord mesenchymal stem cells (HUCMSCs) exhibit stronger attachment than bone marrow-derived stem cells (BMSCs) and adipose-derived stem cells (ADSCs) (Wang and Yan, 2013). ADSCs display higher adipogenic ability, while BMSCs exhibit stronger chondrogenic and osteogenic capacity (Mohamed-Ahmed et al., 2018). Notably, HUCMSCs can differentiate into osteocytes, chondrocytes or adipocytes, displaying stronger chondrogenic and osteogenic properties than BMSCs(Han et al., 2017). Several studies have indicated the potential efficacy of BMSCs in skin burns (Rasulov et al., 2005; Xu et al., 2012), whereas ADSCs might have advantages due to their biological features in enhancing keratinocyte growth and secreting factors that facilitate wound healing (Li et al., 2019; Zhou et al., 2021). However, the limited sources and low survival rate of MSCs *in vivo*, as well as the host immune response towards MSCs, have severely restricted the use of stem cell-based therapy (Nakamura et al., 2007).

#### 1.1.2 Conditioned medium

Emerging evidence suggests that MSCs exert effects by generating a wide range of bioactive factors. The factors are referred to as conditioned medium, consisting of growth factors, hormones, cytokines, chemokines, cell adhesion molecules, lipid mediators, ectosomes and exosomes (Li et al., 2019; Praveen Kumar et al., 2019). MSC-conditioned medium can perform a major role in immune regulation, tissue repair and regeneration and angiogenesis (Tokhanbigli et al., 2019; Lin et al., 2021). Compared with direct MSC transplantation, MSC-conditioned medium is more convenient and safer to use, displaying greater potential in clinical application (Lin et al., 2021).

### 1.2 Biological characteristics of exosomes

#### 1.2.1 Biogenesis

Generally, extracellular vesicles (EVs) are classified into ectosomes and exosomes. Ectosomes (50 nm–1 μm in diameter) are vesicles derived from outwards budding of the plasma membrane and consist of microvesicles, microparticles, and large vesicles. Exosomes (40–160 nm in diameter) are endosomal vesicles produced by double invagination of the plasma membrane (Colombo et al., 2014). The invagination of the plasma membrane generates early-sorting endosomes (ESEs), which then evolve into late-sorting endosomes (LSEs) and eventually form multivesicular bodies (MVBs) with intraluminal vesicles (ILVs). MVBs can be broken down by lysosomes or autophagosomes, or release ILVs as exosomes by fusing with the plasma membrane (He et al., 2018).

#### 1.2.2 Isolation

Currently, various technologies are employed for EV isolation, including 1) differential ultracentrifugation, which is most common and simple but time-consuming (Tauro et al., 2012; Greening et al., 2015); 2) density gradient ultracentrifugation, which is complicated and time-consuming but can isolate exosomes with high purity (Tauro et al., 2012; Greening et al., 2015); 3) size exclusion chromatography, economical and can keep EVs intact but with no specificity for nonexosomal substances and lower yield (Böing et al., 2014); 4) tangential flow filtration, simple and efficient but isolates exosomes with reduced purity (Heinemann et al., 2014; McNamara et al., 2018); 5) affinity capture, highly specific and simple but less efficient and with low yield (Tauro et al., 2012); 6) polyethylene glycol (PEG) precipitation, simple and cost-effective but isolating exosomes with rather low purity (Rider et al., 2016)^;^ and 7) reagent kits such as the exoEasy Maxi kit (QIAGEN), simple but expensive (Zhang et al., 2020). Despite the development of numerous methods for the extraction of exosomes, no standard method for exosome isolation has been established. Therefore, to facilitate the yield and purity, combining multiple extraction methods might be more efficient.

#### 1.2.3 Characterization

Generally, the characterization of isolated exosomes consists of three aspects: 1) detection of the morphological structure of exosomes by scanning electron microscopy (SEM) or transmission electron microscopy (TEM) (Pisitkun et al., 2004); 2) identification of the size and concentration of exosomes by nanoparticle tracking analysis technology (NTA) (Maas et al., 2015) or only the size of exosomes by dynamic light scattering technology (Gercel-Taylor et al., 2012); and 3) detection of negative markers, such as calnexin, and positive markers, including integral exosomal membrane proteins (e.g., CD63, CD9, and CD81) and inner peripheral membrane proteins (e.g., TSG101, ALIX), by Western blotting, enzyme-linked immunosorbent analysis or flow cytometry (Pospichalova et al., 2015; Shao et al., 2018; Théry et al., 2018). The International Society for Extracellular Vesicles (ISEV) suggested that at least one negative and three positive EV protein markers should be detected (Théry et al., 2018).

#### 1.2.4 Function

Exosomes derived from mammals or plants are similar in morphology and immunophenotype and share common biological functions, such as proliferation, migration, adhesion, and apoptosis; however, they present heterogeneous components and characteristics (Wang et al., 2020; Dad et al., 2021). Unlike mammals, plants are free of zoonotic or human pathogens. Therefore, plants derived exosomes exhibit non-immunogenic and innocuous property over mammals derived exosomes, which is also attributed to their efficient uptake by recipient cells and delivery of therapeutic agents, and cost-efficient production (Dad et al., 2021). Up to date, research suggested exosomes regulate processes such as development, immune responses, cardiovascular and metabolic disease, neurodegeneration and cancer (Kalluri and LeBleu, 2020). Exosomes derived from tissue-specific MSC exhibit heterogeneous characteristics and application (Table 1). Compared with MSC, MSC-exosomes display unique advantages, such as easier access and storage, few ethical issues, superior bio-compatibility and intrinsic homing effect, possessing promising therapeutic potential (Zhou et al., 2022).

**TABLE 1:** Heterogeneous characteristics and application of ADSC, BMSC and HUCMSC exosomes.

Exosomes source	Characteristic	Application
ADSC	mass production	wound healing and scar prevention Zhang et al. (2018)
BMSC	easier accessibility	bone and cartilage regeneration, peripheral-nerve recovery ischemia-reperfusion injury He et al. (2020); He et al. (2020); Fan et al. (2022)
HUCMSC	less immunogenic	tissue regeneration, especially skin, angiogenesis Xue et al. (2022)

## 2 Effect of exosomes and conditioned medium on keloids and hypertrophic scars

### 2.1 Therapeutic role of exosomes in keloids and hypertrophic scars

ADSC exosomes are the most widely studied and used intervention in keloid and hypertrophic scar treatment so far. Researchers have found that ADSC exosomes inhibit proliferation and extracellular matrix (ECM) production keloid fibroblast (Li et al., 2022) and hypertrophic scar fibroblast (Yuan et al., 2021). In addition, human amniotic epithelial cell exosomes suppress hypertrophic scar formation (Zhao et al., 2017). Meanwhile, inhibition of lncRNA-ASLNCS5088 and LINC01605 M2 macrophage-derived exosomes impairs fibroblast proliferation, migration and invasion (Chen et al., 2019b; Zhu et al., 2021) (Table 2).

**TABLE 2:** Therapeutic exosome in keloids and hypertrophic scars.

Exosomes source	Source	Molecules	Disease
ADSC	100 μg/mL	Notch 1	Keloid Li et al. (2022a)
ADSC	10,100 μg/mL	-	Keloid Wu et al. (2021)
ADSC	20 μg/mL	miR-192-5p	Hypertrophic scar Li et al. (2021)
ADSC	10 μg/mL	miR-29a	Hypertrophic scar Yuan et al. (2021)
amniotic epithelial cell	100 μg/mL	-	Hypertrophic scar Zhao et al. (2017)
M2 Macrophage	-	ASLNCS5088	Hypertrophic scar Chen et al. (2019)
M2 Macrophage	-	LINC01605	Hypertrophic scar Zhu et al. (2021)

### 2.2 Therapeutic role of conditioned medium in keloids and hypertrophic scars

Exosomes are essential and crucial components of the conditioned medium, rich in signaling molecules such as protein, mRNA, and miRNA, but the isolation methods are complicated, time-consuming, and with low yield and purity. Compared with exosomes, conditioned medium is rich in growth factors, cytokines, chemokines, but the preparation process includes trypsin digestion and *in vitro* culture, adding the risk of biological contamination (Cai et al., 2020). Studies have shown the therapeutic effect of conditioned medium on keloids and hypertrophic scars. It was revealed that ADSC- and Amnion-MSC-conditioned medium attenuated keloid fibroblast activation (Liu et al., 2018; Sato et al., 2018). Human fetal dermal mesenchymal stem cell- and human Wharton’s jelly stem cell-conditioned medium exerted similar effects on keloid fibroblasts (Fong et al., 2014; Jiao et al., 2017). Chyle fat-derived stem cell-conditioned medium prevents hypertrophic scar fibroblast activation (Chen et al., 2019a). Hu et al. (2019) observed that hypertrophic scar formation was inhibited by bone marrow concentrate-induced MSC-conditioned medium(Table 3).

**TABLE 3:** Therapeutic conditioned medium in keloids and hypertrophic scars.

Conditioned medium source	Disease
ADSC	Keloid Liu et al. (2018)
ADSC	Keloid Yang et al. (2021)
ADSC	Keloid Wang et al. (2018)
Amnion-Derived MSC	Keloid Sato et al. (2018)
Fetal dermal MSC	Keloid Jiao et al. (2017)
Wharton’s jelly stem cell	Keloid Fong et al. (2014)
Chyle fat-derived stem cell	Hypertrophic scar Chen et al. (2019a)
BMSC	Hypertrophic scar Hu et al. (2019)
BMSC	Keloid Fang et al. (2016)
Wharton’s jelly stem cell	Keloid Arno et al. (2014)

More importantly, combination therapy assisted conditioned medium in reducing hypertrophic scarring. Botulinum toxin type A combined with mesenchymal stem cell-conditioned medium could effectively treat hypertrophic scars (Hu et al., 2020). In addition, hypertrophic scars could be reduced by combining fractional laser and human umbilical cord mesenchymal stem cell HUCMSC-conditioned medium (Zhang et al., 2022). In addition, hydrogels combined with lyophilized ADSC-conditioned medium reduce scar formation (Zhang et al., 2021). Consistent with previous research, the combination of β-glycerophosphate hydrogel and HUCMSC-conditioned medium prevented the formation of hypertrophic scar tissue (Zhou. et al., 2019) (Table 4).

**TABLE 4:** Combination therapy of stem cell conditioned medium in hypertrophic scars.

Conditioned medium source	Assisted therapy	Disease
MSC	Botulinum toxin type A	Hypertrophic scar Hu et al. 2020
HUCMSC	Fractional laser	Hypertrophic scar Zhang et al. (2022a)
ADSC	Polysaccharide hydrogel	Hypertrophic scar Zhang et al. (2021)
HUCMSC	Thermosensitive Hydrogel	Hypertrophic scar Zhou et al. (2019a)

### 2.3 Pathogenic advances of exosomes in keloids and hypertrophic scars

Exosomes derived from keloids and hypertrophic scars might contribute to the occurrence and development of keloids and hypertrophic scars (Table 5). Keloid fibroblast exosomes release miR-21 and increase cell proliferation and collagen production (Li et al., 2021). Consistently, hypertrophic scar fibroblast-released exosomes promote normal fibroblast proliferation and migration (Cui et al., 2022). Moreover, exosomes derived from keloid patient plasma could enhance normal fibroblast proliferation and fibrogenesis (Hu et al., 2022). Intracellular communication via exosomes between melanocytes and fibroblasts plays a key role in forming scars and keloids. Melanocyte-derived exosome miR-7704 facilitates keloid formation by activating the TGF-β/Smad pathway (SHEN et al., 2022).

**TABLE 5:** Pathogenic role of exosomes in keloids and hypertrophic scars.

Exosomes source	Molecules	Disease
Keloid fibroblasts	miR-21	Keloid Li et al. (2021a)
Hypertrophic scar fibroblasts	TAK1	Hypertrophic scar Cui et al. (2022)
Keloid patient plasma	miR-193a-5p	Keloid Hu et al. (2022)
Melanocyte	miR-7704	Keloid SHEN et al. (2022)

## 3 Possible mechanisms of exosomes in keloids and hypertrophic scars

**FIGURE 1:**
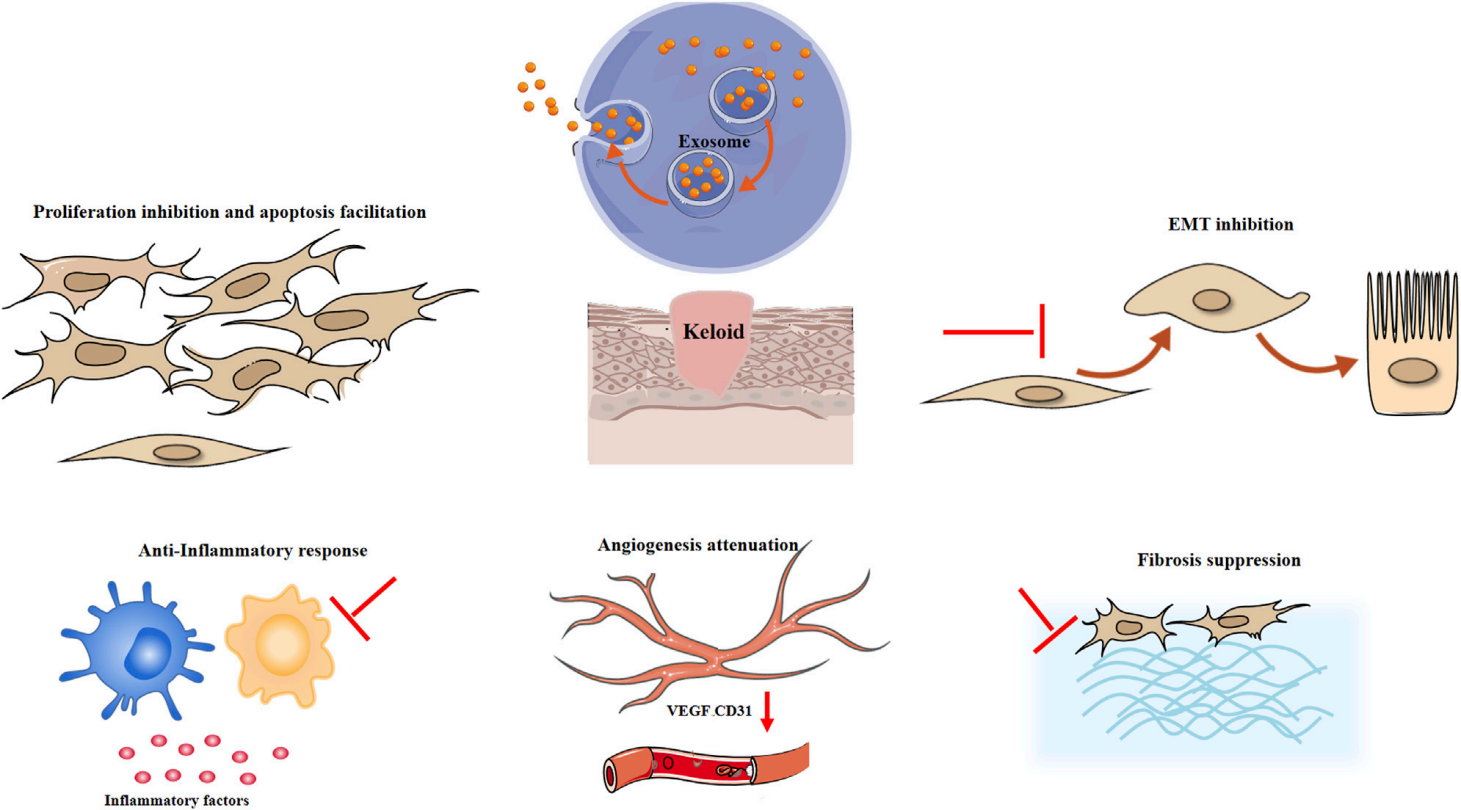
Possible mechanism of exosomes in keloids and hypertrophic scars. EMT epithelial mesenchymal transition, VEGF vascular endothelial growth factor.

Hypertrophic scars and keloids are benign fibroproliferative disorders that may arise after skin injury. Keloids are dermal tumors characterized by abnormal fibroblast proliferation and excessive deposition of extracellular matrix. Clinically, keloids usually manifest as a hard raised scar that extends beyond the boundary of the injury. Hypertrophic scars resemble keloids but exhibit differences in clinical manifestation, histology, and epidemiology. Hypertrophic scars are generally soft, with normal skin color, do not grow beyond the original site of the wound, have low recurrence rates, and histologically exhibit well-organized type III collagen bundles. Keloids exhibit disorganized, large thick, type I and III collagen bundles with no myofibroblast nodules (Gauglitz et al., 2011). Hypertrophic scars and keloids possess common pathological processes to varying degrees, involving proliferation, apoptosis inhibition, fibrosis, angiogenesis, inflammatory response and epithelial mesenchymal transition (EMT) (Limandjaja et al., 2020; Wang et al., 2022), which might indicate the possible therapeutic mechanism of exosomes in keloids and hypertrophic scars (Figure 1).

### 3.1 Proliferation inhibition and apoptosis promotion

ADSC exosomes may attenuate the proliferation and migration and promote the apoptosis of keloid fibroblasts by inhibiting the TGF-β1/Smad pathway (Wu et al., 2021). ADSC-derived exosomes also ameliorated the proliferation and migration of hypertrophic scar fibroblasts (Li et al., 2021). It was shown that MSC-exosomes might facilitate tissue regeneration. However, it was reported that ADSC exosomes facilitate cell growth at 5 and 10 μg/mL (Ren et al., 2022) but suppress cell proliferation at 100 μg/mL (Li et al., 2022). It is hypothesized that the microenvironment and heterogeneity of fibroblasts might be responsible for the dual role of exosomes in tissue synthesis. The wound healing process can be divided into inflammatory phase, proliferative phase and remodelling phase (Monaco and Lawrence, 2003). In the proliferative phases, myofibroblasts are activated, producing ECM components and contracting wound. In the remodelling phase, myofibroblasts secreted matrix metalloproteinase and synthesizing collagen type I. Research suggests that fibroblast in different phase of wound healing display different function and response to growth factors and other molecules (Talbott et al., 2022). Therefore, exosomes might display different role towards fibroblasts, promoting tissue regeneration in the early phase (Hu et al., 2016) and inhibiting excessive ECM synthesis to prevent scar formation in the latter remodelling phase (Wang et al., 2017). However, due to complicated process of wound healing, further research is required to elucidate the role of exosomes in different phase of wounding healing and various subtypes of fibroblasts, such as reticular fibroblasts, papillary fibroblasts and myofibroblasts. In addition, ADSC-conditioned medium suppresses keloid fibroblast growth and facilitates apoptosis through the arachidonic acid-derived cyclooxygenase-2/prostaglandin E2 cascade (Yang et al., 2021). Consistently, human fetal dermal mesenchymal stem cells suppressed the growth of keloid fibroblasts and induced apoptosis by regulating BCL2/BAX protein expression (Jiao et al., 2017). However, BMSC-conditioned medium inhibited hypertrophic scar fibroblast and keloid fibroblast proliferation and migration but did not induce apoptosis (Fang et al., 2016). Similarly, Wharton’s jelly mesenchymal stem cell conditioned medium significantly prevented the growth of keloid fibroblasts, with no significant effect on the apoptosis rate (Arno et al., 2014). Except for exosomes, the conditioned medium includes soluble factors, which might exhibit anti-apoptotic effects.

### 3.2 Fibrosis

Keloids and hypertrophic scars are fibroproliferative diseases characterized by the pathological accumulation of ECM (Andrews et al., 2016; Lian and Li, 2016). A large and growing body of literature has demonstrated that MSC-exosomes display antifibrotic effects on hypertrophic scars and keloid fibroblasts. To determine the antifibrotic effect of ADSC-exosomes, Wang et al. detected the mRNA expression of ECM-related genes in keloid fibroblasts. The results showed that the mRNA levels of PAI-1, TIMP-1, and collagen 1 were significantly inhibited by ADSC-conditioned medium (Wang et al., 2018). Meanwhile, ADSC-derived exosomes may inhibit the proliferation, migration, and collagen synthesis of keloid fibroblasts by inhibiting the TGF-β1/Smad pathway, thus reducing scar formation (Wu et al., 2021). In addition, Yuan et al. (2021) found that miR-29a-modified ADSC-exosome therapy can downregulate the TGF-β2/Smad3 signaling pathway to attenuate collagen deposition and ECM synthesis in hypertrophic scar fibroblasts. It was reported that miR-192-5p prevents hypertrophic scar fibrosis by targeting IL17RA (Li et al., 2021). miR-let-7d mimics effectively ameliorated hypertrophic scar fibrosis (Zhao et al., 2023). Collectively, these studies outlined a crucial antifibrotic role of exosomes in hypertrophic scars and keloid fibroblasts.

### 3.3 Angiogenesis

During the wound healing process, excessive angiogenesis significantly facilitates keloid formation by continuously supplying nutrients, which is similar to tumours (Viallard and Larrivée, 2017; Korntner et al., 2019). Thus, we mainly discussed the effect of exosomes on angiogenesis in tumors. Wang et al. (2018) first demonstrated that ADSC-exosomes disrupted the microvessel structure in keloid tissue explants, with reduced CD31^+^ and CD34^+^ vessels. Similarly, it was reported that BMSC exosome-derived miR-16 could attenuate angiogenesis and tumor progression by directly targeting vascular endothelial growth factor (VEGF) in breast cancer (Lee et al., 2013). In addition, BMSC exosome-derived miR-100 inhibited VEGF expression via the mTOR/HIF-1α pathway, thereby suppressing the angiogenesis of breast cancer (Pakravan et al., 2017). However, it was revealed that MSC-exosomes activated the extracellular signal-regulated kinase 1/2 (ERK1/2) pathway, thereby elevating VEGF expression and eventually contributing to tumor angiogenesis (Zhu et al., 2012). Overall, these studies suggested a critical role of MSC-exosomes in angiogenesis.

### 3.4 Inflammatory response

Excessive inflammation in the wound healing phase causes abnormal scarring that contributes to a range of abnormal phenotypes, such as hypertrophic and keloid scars (Ogawa, 2017). Studies have revealed that macrophages, mast cells, dendritic cells (DCs) and regulatory T cells are involved in the occurrence of keloids (Zhang et al., 2006; Onodera et al., 2007; Murao et al., 2014; Direder et al., 2022). MSC-exosomes exert immunomodulatory effects and alleviate the inflammatory response by suppressing immune cell function and the synthesis of inflammatory cytokines (Harrell et al., 2019). M2 macrophage polarization and regulatory T-cell expansion were induced by MSC-exosomes (Cho et al., 2018; Sun et al., 2022). MSC-exosomes could decrease the number of mast cells in skin lesions and the maturation of bone marrow DCs, alleviating DC-induced immune responses (Shahir et al., 2020). BMSC exosomes prevented the growth and induced the apoptosis of CD4^+^ T cells (Del Fattore et al., 2015). The proliferation and differentiation of B lymphocytes was inhibited by BMSC exosomes (Conforti et al., 2014). In microglial cells treated with MSC-exosomes, the production of inflammatory cytokines (TNFα and IL-1β) was inhibited, while the generation of anti-inflammatory cytokines (IL-10 and TGF-β) was enhanced (Harrell et al., 2019). These findings suggested that MSC-exosomes exhibit anti-inflammatory effects by transforming proinflammatory immune cells (M1 macrophages, DCs, CD4^+^ T cells) into anti-inflammatory M2 macrophages, tolerogenic DCs and regulatory T cells.

### 3.5 EMT

EMT is a cellular process in which epithelial cells acquire a mesenchymal phenotype, elevating invasiveness. Epithelial-mesenchymal transition plays a role in the development of hypertrophic scars and keloids (Dongre and Weinberg, 2019; Xia et al., 2022). Numerous studies have revealed that MSC-derived EVs ameliorate EMT. It was found that MSC-exosomes alleviated the EMT of radiation-induced alveolar epithelial cells (Li et al., 2022). HUCMSC-derived exosomes suppressed EMT in cholangiocarcinoma (Li and Wang, 2022) and significantly downregulated colorectal cancer cell EMT via the miR-100/mTOR/miR-143 pathway (Jahangiri et al., 2022). In addition, the EMT of breast cancer cells was restrained by BMSC-derived exosomes (Zhang et al., 2022). In contrast, Shi et al. (2016) found that BMSC-derived exosomes enhanced the EMT of nasopharyngeal carcinoma cells. Similarly, Zhou et al. (2019) observed that HUCMSC-EVs induced EMT by activating the ERK pathway, contributing to breast cancer development and metastasis. These findings collectively suggested that exosomes might exhibit a dual role in EMT, and their specific role in keloids and hypertrophic scars needs to be explored in the future.

## 4 Conclusion

In summary, MSC-exosomes exhibit multiple effects on keloid and hypertrophic scar formation and progression and function as a promising clinical cell-free therapy. Differences in exosome dose and source might explain its dual role in keloids and hypertrophic scars. To date, researchers have only explored the role of MSC-exosomes in keloids and hypertrophic scar fibroblasts. In contrast, the impact of MSC-exosomes on keloid and hypertrophic scar keratinocytes and immune cells remains unknown. In addition, the clinical application and combination therapy of MSC-exosomes in keloid and hypertrophic scar treatment is still at the initial stage. Therefore, further research is required to elucidate their molecular mechanism and facilitate clinical application.
